# Ventricular Dyssynchrony and Function Improve following Catheter Ablation of Nonseptal Accessory Pathways in Children

**DOI:** 10.1155/2013/158621

**Published:** 2013-06-18

**Authors:** Sylvia Abadir, Anne Fournier, Marc Dubuc, Georgia Sarquella-Brugada, Patrick Garceau, Paul Khairy

**Affiliations:** ^1^Department of Pediatric Cardiology, Sainte-Justine Hospital, Université de Montréal, Montreal, QC, Canada H3T 1C5; ^2^Department of Cardiology, Montreal Heart Institute, Université de Montréal, Montreal, QC, Canada H1T 1C8

## Abstract

*Introduction*. Paradoxical or hypokinetic interventricular septal motion has been described in patients with septal or paraseptal accessory pathways. Data regarding nonseptal pathways is limited. *Methods and Results*. We quantified left ventricular dyssynchrony and function in 16 consecutive children, 14.2 ± 3.7 years, weighing 53 ± 17 kg, prior to and following catheter ablation of bidirectional septal (*N* = 6) and nonseptal (*N* = 10) accessory pathways. Following ablation, the left ventricular ejection fraction increased by 4.9 ± 2.1% (*P* = 0.038) from a baseline value of 57.0% ± 7.8%. By tissue Doppler imaging, the interval between QRS onset and peak systolic velocity (Ts) decreased from a median of 33.0 ms to 18.0 ms (*P* = 0.013). The left ventricular ejection fraction increased to a greater extent following catheter ablation of nonseptal (5.9% ± 2.6%, *P* = 0.023) versus septal (2.5% ± 4.1%, *P* = 0.461) pathways. The four patients with an ejection fraction <50%, two of whom had left lateral pathways, improved to >50% after ablation. Similarly, the improvement in dyssynchrony was more marked in patients with nonseptal versus septal pathways (difference between septal and lateral wall motion delay before and after ablation 20.6 ± 7.1 ms (*P* = 0.015) versus 1.4 ± 11.4 ms (*P* = 0.655)). *Conclusion*. Left ventricular systolic function and dyssynchrony improve after ablation of antegrade-conducting accessory pathways in children, with more pronounced changes noted for nonseptal pathways.

## 1. Introduction

Manifest atrioventricular accessory pathways in the so-called Wolff-Parkinson-White syndrome result in electrical and mechanical preexcitation of ventricular myocardium at their site of insertion. Earlier echocardiographic studies demonstrated that left posteroseptal accessory pathways are associated with abnormal left ventricular posterior wall motion [1–3] and that right anterior or right posteroseptal pathways produce abnormal interventricular septal motion [2, 4–6]. Since these studies predated the era of catheter ablation, localization of accessory pathways was based solely on electrocardiographic criteria [7]. More recent reports underscored the potential for right-sided and septal pathways [8, 9] to result in left ventricular dysfunction that improves following catheter ablation [10–12]. However, it is generally believed that left lateral accessory pathways produce a lesser degree of ventricular dyssynchrony and dysfunction by virtue of their location and relative distance away from the normal conduction system. There is a paucity of data regarding potential deleterious effects of nonseptal pathways on inter- and intraventricular dyssynchrony. We, therefore, sought to characterize and compare the degree of left ventricular dyssynchrony, systolic, and diastolic functions by detailed echocardiographic analyses with tissue Doppler imaging prior to and following catheter ablation of septal and nonseptal accessory pathways.

## 2. Materials and Methods

### 2.1. Study Population

We enrolled consecutive children (≤18 years of age) with a diagnosis of Wolff-Parkinson-White syndrome (i.e., manifest ventricular preexcitation associated with palpitations) referred for catheter ablation at Sainte-Justine Hospital over a 2-year period. We excluded patients with a congenital heart defect (other than an isolated bicuspid aortic valve) and those in whom transthoracic echocardiograms could not be performed for logistical reasons prior to or following catheter ablation. The protocol was approved by the local institutional review board, and written informed informed consent was obtained from all patients.

### 2.2. Electrocardiograms and Transthoracic Echocardiography

Electrocardiograms were performed prior to and after catheter ablation in all patients. PR intervals were measured by computed analyses. The accessory pathway location was initially classified by an established electrocardiographic algorithm in children [13] and subsequently refined according to the site of successful catheter ablation. Two-dimensional transthoracic echocardiography was performed using standard parasternal long axis, apical 4-chamber, and subcostal views prior to and within 24 hours after catheter ablation. Cardiac function, valve regurgitation, and dyssynchrony parameters were assessed. For each parameter, three consecutive cardiac cycles were recorded and the three values averaged. Two independent reviewers analyzed offline transthoracic echocardiographic data.

**Figure 1:**
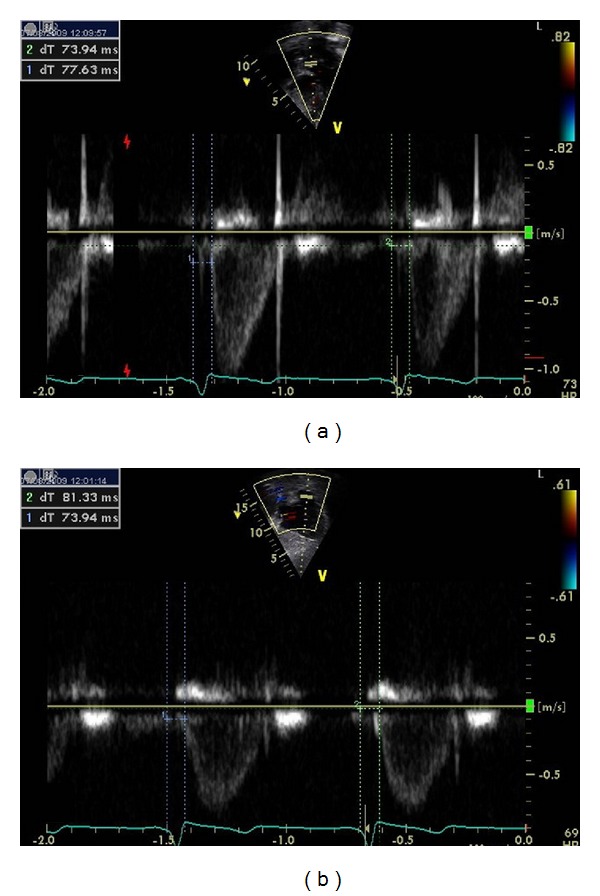
Determination of interventricular mechanical delay (IVMD) using pulsed Doppler mode, corresponding to the difference between aortic (a) and pulmonary (b) preejection delay. No significant delay in left ventricular contraction is observed.

**Figure 2:**
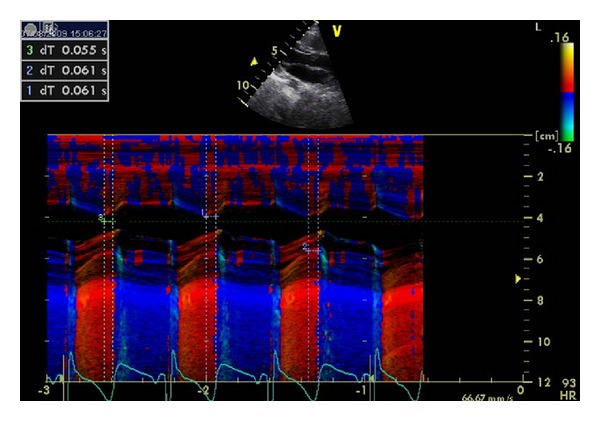
Assessment of septal-to-posterior wall motion delay (SPWMD) using M-mode imaging. Normal SPWMD (<130 ms) is demonstrated.

**Figure 3:**
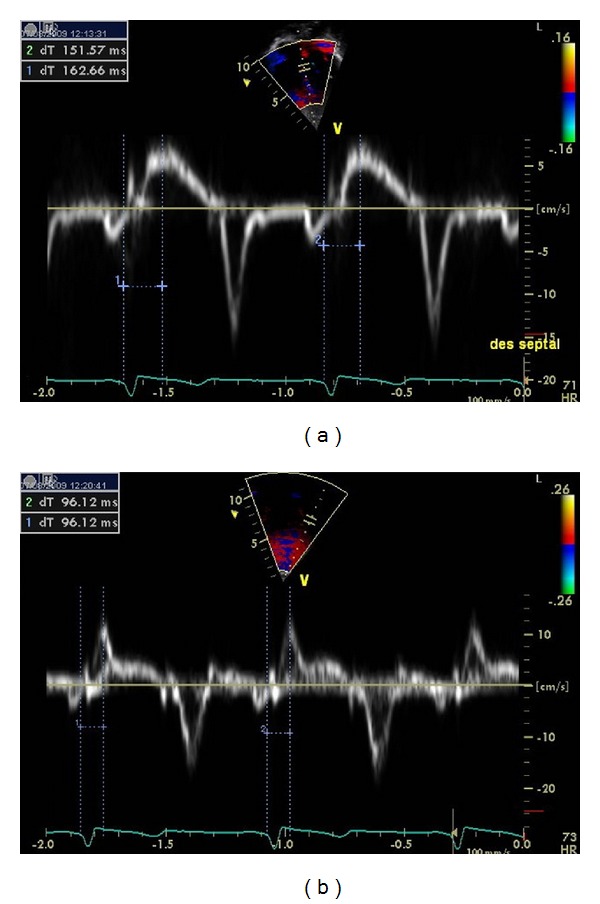
Determination of the delay between time-to-peak systolic velocity at basal septal (a) and basal lateral (b) segments using tissue Doppler imaging. A significant delay is demonstrated in this patient (no. 10).

**Figure 4:**
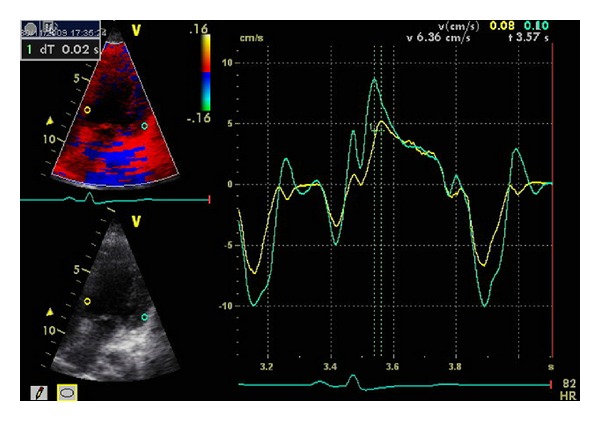
Determination of time-velocity curves using color-coded tissue Doppler and Q-analyses (from left ventricular septal and lateral wall sample points selected in an apical long-axis view). In this example (patient no. 6), no significant delay is demonstrated between septal and lateral left ventricular walls following catheter ablation.

Cardiac dyssynchrony was assessed by the following criteria (Figures 1, 2, 3, and 4):

- aortic preejection delay (APD) using M-mode and pulsed Doppler modes, measured from the onset of the QRS complex to the beginning of aortic ejection (abnormal if ≥140 ms) [14];
- interventricular mechanical delay (IVMD) using the pulsed Doppler mode, defined as the difference between aortic and pulmonary preejection delay (abnormal if ≥40 ms) [14];
- septal-to-posterior wall motion delay (SPWMD) using M-mode imaging (abnormal if ≥130 ms) [15];
- delay between time to peak systolic velocity at basal septal and basal lateral segments using tissue Doppler imaging (abnormal if ≥60 ms) [16]. This measure was first performed using pulsed tissue Doppler and then confirmed using color-coded tissue Doppler and time-velocity curves (from left ventricular septal and lateral wall sample points selected in an apical long-axis view).

The left ventricular ejection fraction was quantified using the Simpson method [17] in the apical view and considered abnormal if ≤55%. Aortic and mitral valve regurgitation was assessed semiquantitatively by 2D color Doppler imaging and classified as discrete, mild, moderate, or severe [18]. Diastolic parameters were assessed using the pulsed Doppler mode, tissue Doppler imaging (E wave velocity, E wave deceleration time (EDT), and the E/E′ ratio at basal septal and basal lateral left ventricular segments). Diastolic function was considered abnormal if the EDT was <150 or >220 ms and if E/E′ > 15 [19–21]. Complications such as pericardial effusion and increased valvular insufficiency were assessed by the postprocedural transthoracic echocardiogram.

### 2.3. Catheter Ablation Procedure

All catheter ablation procedures were performed under general anaesthesia. Three quadripolar catheters were advanced to the high right atrium, His position, and right ventricular apex. A decapolar catheter was placed in the coronary sinus. An initial electrophysiological study was performed to assess baseline intervals, atrial, atrioventricular nodal, accessory pathway, ventricular refractory periods, Wenckebach physiology, retrograde conduction, and inducible supraventricular tachycardia. The atrial insertion of the accessory pathway was identified as the earliest atrial signal during retrograde accessory pathway conduction. The ventricular insertion was assessed during maximum preexcitation as the site of earliest ventricular depolarization. Accessory pathways were ablated using transcatheter cryoenergy for septal and radiofrequency energy for lateral pathways (left or right). Coexisting arrhythmias, such as atrioventricular nodal reentrant tachycardia, were ablated during the same intervention. Catheter ablation was considered successful if, after 30 minutes of observation, there was no evidence of antegrade or retrograde conduction across an accessory pathway and no inducible supraventricular tachycardia with and without an isoproterenol infusion (0.02 *μ*g/kg/min). Barring any complication, patients were discharged the following day.

### 2.4. Statistical Analysis

Continuous variables are summarized by mean ± standard deviation or median and interquartile range (IQR; 25th, 75th percentiles) depending on normality of distribution. Categorical variables are represented by frequencies and percentages. Intrapatient comparisons prior to and following ablation were assessed by paired Student's *t*-tests or Wilcoxon signed-rank tests. Two-tailed *P* values <0.05 were considered statistically significant. Analyses were performed with SAS software Version 9.2 (SAS Institute, Cary, NC, USA). 

## 3. Results

### 3.1. Patient and Procedural Characteristics

Overall, 16 children (5 females), age 14.2 ± 3.7 years, weight 53 ± 17 kg were systematically assessed. All had Wolff-Parkinson-White syndrome, structurally normal hearts, and evidence of sustained supraventricular tachycardia by Holter monitoring or external event loop recorders. Patient and procedural characteristics are summarized in Table 1. Septal or paraseptal pathways were present in 6 (37.5%) patients and nonseptal pathways in 10 (62.5%). The nonseptal pathways consisted of  left lateral (*N* = 5), right lateral/anterolateral (*N* = 3), and left posterior (*N* = 2) connections. All patients underwent successful catheter ablation. Cryoenergy was employed in 4 patients and radiofrequency energy in 12.

### 3.2. Echocardiographic Parameters

**Figure 5:**
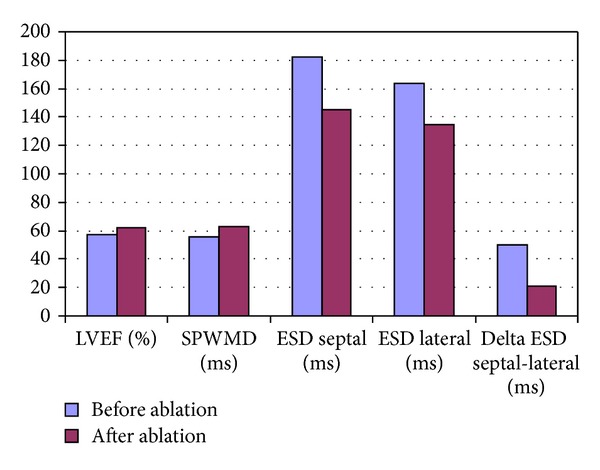
Echocardiographic parameters prior to and following catheter ablation. LVEF denotes left ventricular ejection fraction; SPWMD, septal-to-posterior wall motion delay; ESD, electrosystolic delay (or time-to-peak systolic velocity).

Prior to ablation, no differences in left ventricular ejection fraction or indices of left ventricular dyssynchrony were observed in patients with septal versus nonseptal accessory pathways.  Table 2 and Figure 5 summarize the echocardiographic parameters prior to and following catheter ablation. Prior to ablation, 6 patients had a left ventricular ejection fraction <55% and 4 <50%. Following ablation, the ejection fraction increased by 4.9 ± 2.1% (*P* = 0.038) from a baseline value of 57.0 ± 7.8%. This was accompanied by a significant reduction in the difference between aortic and pulmonary preejection times (11.0 ± 3.3 ms, *P* = 0.017). The left ventricular ejection fraction increased to a greater extent following ablation of nonseptal (5.9 ± 2.6%, *P* = 0.023) versus septal (2.5 ± 4.1%, *P* = 0.461) pathways. The 4 patients with a left ventricular ejection fraction <50% prior to ablation, 2 of whom had left lateral pathways, improved to >50% after ablation. By tissue Doppler imaging, the time to peak systolic velocity decreased from 33.0 ms (IQR 20.0, 48.0) to 18.0 ms (IQR 5.0, 24.0), *P* = 0.013. No significant change in septal-to-posterior wall motion delay or diastolic parameters was noted.

Similar to changes in left ventricular ejection fraction, the magnitude of improvement in left ventricular dyssynchrony was more marked in patients with nonseptal versus septal pathways. The difference between septal and lateral wall motion delay before and after ablation was 20.6 ± 7.1 ms (*P* = 0.015) versus 1.4 ± 11.4 ms (*P* = 0.655) for nonseptal versus septal pathways, respectively. 

## 4. Discussion

A possible pathophysiological link between accessory pathways and ventricular dysfunction has been proposed in the absence of tachycardia-induced cardiomyopathy [9, 12, 22]. Most prior reports investigating this association have focused on septal pathways [9–12]. In our study, which incorporated detailed echocardiographic assessment prior to and following catheter ablation of accessory pathways, the following salient features were noted: (1) left ventricular dyssynchrony and dysfunction were observed in patients with septal and nonseptal pathways; (2) dyssynchrony and dysfunction were reversible following successful ablation of the accessory pathway; (3) improvements were more marked in patients with nonseptal pathways; and (4) left ventricular diastolic function parameters prior to ablation (i.e., mean lateral and septal E/E′ values of 5.6 and 7.1, resp.) were comparable to a cohort of 325 healthy children [23] and unchanged following ablation.

Tomaske et al. retrospectively reported a series of 34 children (mean age 14.2 years) with Wolff-Parkinson-White syndrome who underwent successful radiofrequency ablation of right septal or posteroseptal accessory pathways [12]. Prior to ablation, 56% of patients had a left ventricular ejection fraction <55%. It improved significantly after catheter ablation, from a mean of 50% to 56%. Classic dyssynchrony parameters were studied. Whereas SPWMD decreased significantly after ablation, no change in IVMD was observed. Five patients underwent two-dimensional strain analysis, which showed a reduction in the difference between the earliest and latest negative strain after ablation. Our study extends these findings to include an analysis of tissue Doppler imaging to children with manifest ventricular preexcitation regardless of its location. 

In another series of 63 children with Wolff-Parkinson-White syndrome (mean age at diagnosis 5.9 years), left ventricular dysfunction was reported only in association with septal accessory pathways [11]. The mean left ventricular ejection fraction was 53% in patients with septal accessory pathways (*N* = 15) compared to 62% for all other locations. Moreover, septal dyskinesia was identified in 6 patients with right septal accessory pathways and was the only factor significantly associated with a reduction in left ventricular ejection fraction. After ablation, the left ventricular ejection fraction significantly improved in these 6 patients from 42 ± 5% to 55 ± 7%. Tissue Doppler imaging was not systematically performed. 

The relationship between dyskinetic segments and left ventricular dysfunction is also supported by the pacemaker literature, which suggests that right ventricular apical pacing may have a detrimental effect on left ventricular function [24–26]. Restoration of cardiac synchrony by cardiac resynchronization therapy is a well-established intervention that improves all-cause mortality [27, 28]. Animal experiments have shown that asynchronous electrical activation induced by ventricular pacing results in asymmetrical hypertrophy and ventricular dilation due to regional differences in workload [29]. A pathophysiological link between nonseptal accessory pathways and ventricular dyssynchrony is supported by such preclinical experiments, which demonstrated that early activation of the left ventricular free wall in paced dogs resulted in significant thinning of left ventricular myocardium and thickening of the late-activated septum. Dyskinetic segments may function much like aneurysms and induce adverse remodelling with progressive dilatation [9, 10]. Hemodynamic changes with altered blood flow patterns may ensue, producing endomyocardial fibroelastic thickening [10]. 

It may, therefore, be hypothesized that ventricular preexcitation can result in remodelling changes similar to ventricular pacing, with segmental dyskinesia leading to thinning, dyssynchrony, ventricular dilation, and, ultimately, ventricular dysfunction. Such a pattern may be produced from any site of ventricular activation remote from the normal conduction system. Our results are consistent with the hypothesis that dyssynchrony induced by nonseptal pathways may be as deleterious, and sometimes more so, than septal pathways. Further studies are required to confirm these findings and to assess the relevance of targeting accessory pathways based solely on hemodynamic effects of preexcitation [30].

*Limitations. *As is often the case with pediatric case series, the study was limited by the small number of patients. Nevertheless, to minimize selection bias, all consecutive children referred for catheter ablation of Wolff-Parkinson-White syndrome were enrolled over a 2-year period. In determining dyssynchrony, echocardiographic assessment was limited to basal septal and lateral segments by means of tissue Doppler imaging. The comparative value of sampling additional segments or the use of potentially complementary imaging techniques, such as strain and strain rate, could not be assessed.

## 5. Conclusion

In a series of pediatric patients with Wolff-Parkinson-White syndrome and absence of structural heart disease, left ventricular systolic function and dyssynchrony improved after successful ablation of antegrade-conducting accessory pathways. The more pronounced changes were observed in patients with nonseptal pathways.

## Figures and Tables

**Table 1:** Baseline and procedural characteristics.

Patient	Age at ablation (years)	Weight (kg)	Body surface area (m^2^)	Energy source	Location of AP	Delta-HV interval (ms)	F/U after ablation (months)
1	16.7	58.7	1.8	RF	LL	77	27.7
2	19.4	73.0	1.9	RF	RPS	53	20.8
3	15.0	58.6	1.7	RF	RPS	40	20.8
4	18.9	62.0	1.7	RF	RPS	10	21.5
5	14.9	56.0	1.6	RF	RPL	45	26.4
6	13.7	54.0	1.6	RF	LPS	18	0.4
7	16.5	77.0	1.9	RF	LP	81	3.9
8	13.0	39.0	1.3	Cryo	RA	23	4.0
9	14.1	71.0	1.8	RF	LL	21	4.0
10	9.7	30.0	1.1	RF	LL	17	4.1
11	7.6	23.0	0.9	Cryo	RAS	N/A	4.2
12	6.8	22.0	0.9	Cryo	RA	40	5.9
13	17.1	60.0	1.7	RF	LL	24	6.5
14	16.4	70.0	1.8	RF	RL	39	9.3
15	15.3	58.0	1.6	RF	LL	19	15.1
16	11.8	40.0	1.3	Cryo	RPS	44	18.5

Summary	14.2 ± 3.7	53.3 ± 17.5	1.5 ± 0.3	RF 12; Cryo 6	6 septal; 10 nonseptal	36.7 ± 21.4	12.1 ± 9.2

AP denotes accessory pathway; F/U: followup; RF: radiofrequency; Cryo: cryoenergy; LL: left lateral; RPS: right posteroseptal; RPL: right posterolateral; LPS: left posteroseptal; LP: left posterior; RAS: right anteroseptal; RAL: right anterolateral; RA: right anterior; RL: right lateral; N/A: not available.

**Table 2:** Echocardiographic parameters prior to and following catheter ablation.

Patient	Location of AP	LV ejection fraction (%)		SPWMD (ms)		ESD septal (ms)		ESD lateral (ms)		Delta ESD septal-lateral (ms)	
		PRE	POST	PRE	POST	PRE	POST	PRE	POST	PRE	POST
1	LL	48	58	101	100	256	143	240	120	16	23
2	RPS	72	68	47	64	164	147	147	120	17	27
3	RPS	55	N/A	58	40	N/A	N/A	N/A	N/A	N/A	N/A
4	RPS	61	57	30	33	211	154	N/A	N/A	N/A	N/A
5	RPL	50	76	53	47	200	110	167	110	33	0
6	LPS	60	63	50	87	157	112	122	92	35	20
7	LP	61	61	5	64	175	135	146	117	29	18
8	RA	48	53	31	40	184	188	280	271	96	83
9	LL	62	59	33	63	125	124	112	122	13	2
10	LL	65	68	44	61	162	180	96	175	66	5
11	RAS	49	62	76	78	293	195	183	N/A	110	N/A
12	RA	52	55	99	10	192	170	133	155	59	15
13	LL	68	77	15	115	140	127	110	103	30	24
14	RL	55	62	80	61	155	N/A	280	N/A	125	N/A
15	LL	49	50	100	72	133	108	110	96	23	12
16	RPS	N/A	62	77	75	N/A	N/A	N/A	N/A	N/A	N/A

Mean ± SD	—	57 ± 8	62 ± 8	56 ± 30	63 ± 26	182 ± 47	145 ± 30	164 ± 64	135 ± 52	50 ± 38	21 ± 23

LV denotes left ventricle; SPWMD: septal-to-posterior wall motion delay; ESD: electrosystolic delay (or time-to-peak systolic velocity); PRE: preablation; POST: postablation; LL: left lateral; RPS: right posteroseptal; RPL: right posterolateral; LPS: left posteroseptal; LP: left posterior; RAS: right anteroseptal; RAL: right anterolateral; RA: right anterior; RL: right lateral; N/A: not available.
